# The effect of hyperoxia on mortality in critically ill patients: a systematic review and meta analysis

**DOI:** 10.1186/s12890-019-0810-1

**Published:** 2019-02-26

**Authors:** Yue-Nan Ni, Yan-Mei Wang, Bin-Miao Liang, Zong-An Liang

**Affiliations:** 1 Department of Respiratory and Critical Care, West China School of Medicine and West China Hospital, Sichuan University, No.37 Guoxue Alley, Chengdu, 610041 China; 2 Department of Respiratory Medicine, Sichuan Second Hospital of Traditional Chinese Medicine, Chengdu, 610031 Sichuan China

**Keywords:** Hyperoxia, Mortality, Meta-analysis

## Abstract

**Background:**

Studies investigating the role of hyperoxia in critically ill patients have reported conflicting results. We did this analysis to reveal the effect of hyperoxia in the patients admitted to the intensive care unit (ICU).

**Methods:**

Electronic databases were searched for all the studies exploring the role of hyperoxia in adult patients admitted to ICU. The primary outcome was mortality. Random-effect model was used for quantitative synthesis of the adjusted odds ratio (aOR).

**Results:**

We identified 24 trials in our final analysis. Statistical heterogeneity was found between hyperoxia and normoxia groups in patients with mechanical ventilation (I^2^ = 92%, *P* < 0.01), cardiac arrest(I^2^ = 63%, *P* = 0.01), traumatic brain injury (I^2^ = 85%, *P* < 0.01) and post cardiac surgery (I^2^ = 80%, *P* = 0.03). Compared with normoxia, hyperoxia was associated with higher mortality in overall patients (OR 1.22, 95% CI 1.12~1.33), as well as in the subgroups of cardiac arrest (OR 1.30, 95% CI 1.08~1.57) and extracorporeal life support (ELS) (OR 1.44, 95% CI 1.03~2.02).

**Conclusions:**

Hyperoxia would lead to higher mortality in critically ill patients especially in the patients with cardiac arrest and ELS.

**Electronic supplementary material:**

The online version of this article (10.1186/s12890-019-0810-1) contains supplementary material, which is available to authorized users.

## Background

Oxygen supplement is a life saving treatment commonly used in the critically ill patients [1]. Excess oxygen delivery was reported to be a very common phenomenon, in which about 50% of the patients showed hyperoxemia and 4% in severe hyperoxemia [2].

Animal studies showed that hyperoxia is associated with adverse events such as histopathological injury, interstitial fibrosis, atelectasis, tracheobronchitis, alveolar protein leakage and infiltration by neutrophils [3]. Hyperoxia interacts with mechanical stretch to augment ventilator-induced lung injury [4]. Moreover, hyperoxia could also lead to a decline of cardiac output, [5] coronary blood flow and myocardial oxygen consumption, [6] and generate free radical-mediated damages in various organs [7]. Studies in human demonstrated that hyperoxia could impair the responsiveness of host defense to infections [8]. Hyperoxia may affect a variety of patients’ biological systems, such as antioxidant enzymes [9] and cytokine production [10] through excessive production of reactive oxygen species. Exaggerated apoptosis, in part through the death receptor-mediated signals, accelerates hyperoxia-induced acute lung injury [11]. However, clinical studies testing the relationship between hyperoxia and mortality in critically ill patients have yielded conflicting results. For example, in a study of 36,307 patients admitted to the intensive care units (ICU), no difference in mortality was noted between the patients exposed to hyperoxia and those who did not [12]. In contrast, Page et al. found that there was an association between hyperxia and increased mortality (adjusted odds ratio[aOR] 1.95, 95% confidence interval[CI] 1.34–2.85) [13]. These conflicts were also seen in some specified diseases such as 1) patients with cardiac arrest, in which Elmer et al. showed a decreased survival (aOR 0.83, 95% 0.69–0.99, *P* = 0.04), [14] while Ihle et al. did not find any difference; [15] and 2) patients after traumatic brain injury(TBI), in which Asher et al. found a reduced mortality by hyperoxia while a contrary result was showed by Davis et al. [16, 17].

Although previous studies have performed the analysis of the relationship between hyperoxia and mortality, no solid conclusion has been drawn. As for several new studies in this topic being published recently, we conducted a systematic review and meta-analysis of all published trials aiming for identifying the roles of hyperoxia in the outcomes of patients in ICU.

## Methods

### Search strategies

Using the keywords of “hyperoxia” or “oxygenation target” or “hyperoxemia” or “oxygen saturation” or “arterial oxygen” and “critically ill” or “intensive care” or “mechanical ventilation”, we conducted a comprehensive computer search in Pubmed, Embase, Medline, Cochrane Central Register of Controlled Trails (CENTRAL) and Information Sciences Institute (ISI) Web of Science from 1946 to December 2016 regardless of publication types or language. All the references listed in the identified articles were reviewed and manually searching for related articles was conducted in order to identify all eligible studies and achieve minimal publication bias.

### Inclusion and exclusion criteria

The including criteria was as follows: 1) the subjects enrolled in each study included patients admitted to ICUs; 2) patients were divided into hyperoxia group and normoxia group; and 3) outcomes contained but not limited to mortality. We excluded studies if the patients were: 1) less than 18 years old; 2) chronic pulmonary disease; 3) acute lung injury or acute respiratory distress syndrome; and 4) in perioperative phase. Animal studies and studies published as reviews or case reports were also excluded.

### Study selection

First of all, two independent authors screened the titles and abstracts. Secondly, after reviewing full texts, the authors included eligible studies according to the previously designed study inclusion criteria. A third author would deal with the disagreement between the above two authors by mutual consensus.

### Data extraction

Recommended by Cochrane [18], two authors independently extracted and recorded desirable information of each enrolled study, which consisted of authors, publication year, study design, country, population, NCT No., primary disease, definition of hyperoxia and comparing group, outcome measures, and study results. For any missing data information, we made attempt to contact the corresponding authors by email for full original data. A third author was consulted when disagreement presence between the two authors.

### Statistical analysis

Data was analyzed in Stata software by an independent statistician. At first, χ^2^ test was used to detect clinical, methodological and statistical heterogeneities. *P* < 0.1 and I^2^ > 50% was used to indicate significant heterogeneity. *Mann-Whitney U-test* was conducted to verify hypothesis and rendered statistical significance as a Z-value and *P*-value < 0.05. Forest plots are used to illustrate the results. Random-effects models were applied in the presence of statistical heterogeneity. The calculation of the effect size was based on the OR between hyperoxia and mortality. The sensitivity analysis was performed to substitute alternative decisions or ranges of values for decisions that were arbitrary or unclear.

## Results

**Fig. 1:**
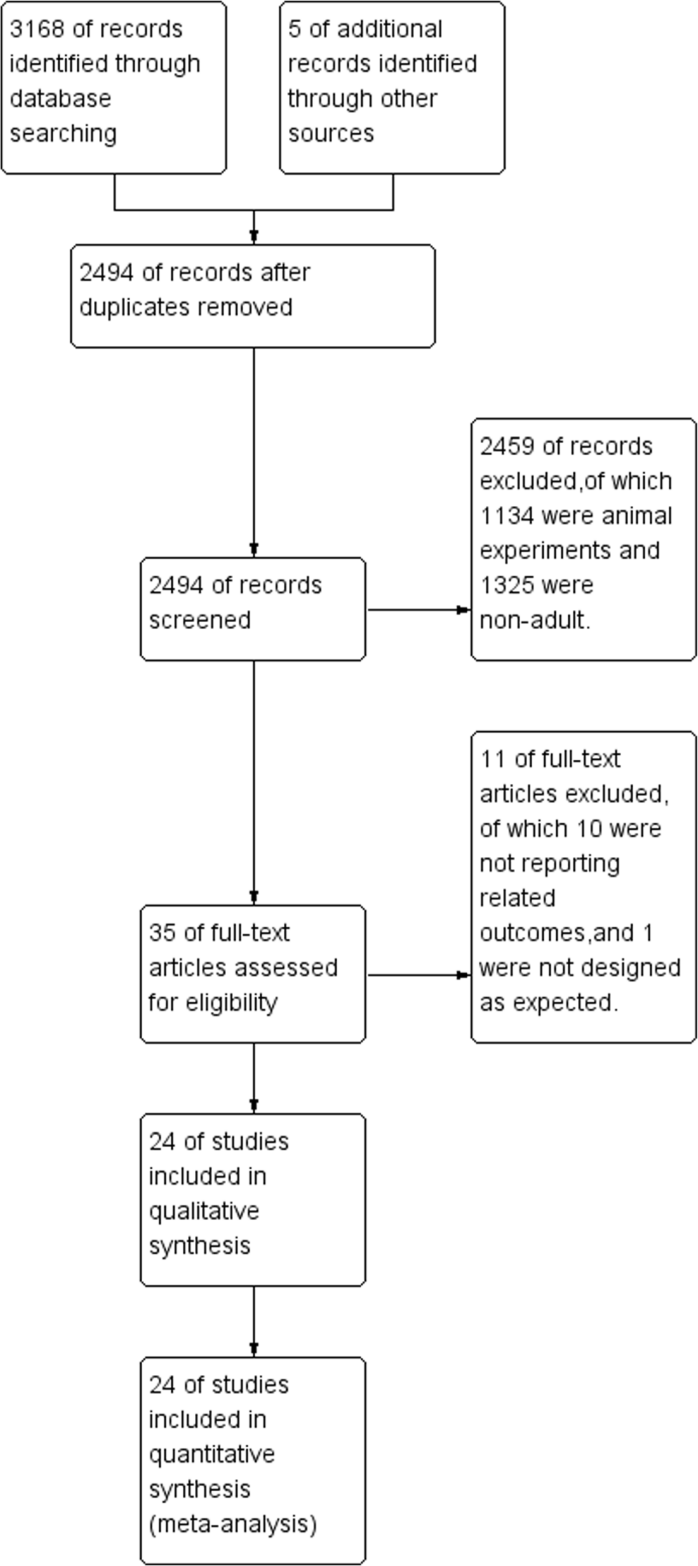
Study flow

Initially 3173 records were identified, of which 3168 were extracted from electronic databases and 5 were from the reference lists review. (Figure 1) By screening the titles and abstracts, 3138 studies were discarded for duplication (*n* = 679), animal experiments (*n* = 1134) and non-adult patients (*n* = 1325). We searched the full-text articles for the remaining 35 studies, and eventually 24 trials were enrolled in our final analysis due to 10 studies not reporting related outcomes, and 1 did not designed as expected.

Hyperoxia defined as partial arterial pressure of oxygen (PaO_2_) > 487 mm Hg(mmHg), 341 mmHg, 300 mmHg, 200 mmHg, 156.7 mmHg, 150 mmHg, 120 mmHg and 100 mmHg was reported in 1, 1, 8, 2, 2, 2, 2, and 3 studies, respectively. One study defined hyperoxia as FiO_2_ of 1.0, and the other two studies did not specify the definition of hyperoxia. As for the definition of normoxia, 12 studies did not report the details and the details of the other 12 studies have been listed in the Table 1.

**Table 1:** Basic characteristics of enrolled studies

Study	Study design	Country	population	NCT.no	Disease	PaO2/ABG	Time of assessment	Cutoff value	Comparator group	Outcome measure reported
Asfar 2017 [36]	Multicenter randomised study	France	442	NCT 01722422	Septic shock	During first 24 h	First 24 h	FiO_2_ 1.0	SaO_2_ 88~95%	28-day mortality
Asher 2013 [16]	Retrospective study	USA	193	NR	traumatic brain injury	HighestPaO_2_	First 72 h	≧200 mmHg	Not exposed to hyperoxia	In-hospital mortality
Bellomo 2011 [20]	Retrospective study	Australia	12,108	NR	cardiac arrest	Worst PaO_2_	First 24 h	≧300 mmHg	Normoxia	In-hospital mortality
Brenner 2012 [25]	Retrospective study	USA	1547	NR	traumatic brain injury	Mean PaO_2_	First 24 h	> 200 mmHg	Normoxia	In-hospital mortality
Davis 2009 [17]	Retrospective study	USA	3420	NR	traumatic brain injury	First PaO_2_	On arrival	> 487 mmHg	Not exposed to hyperoxia	In-hospital mortality
de Jonge 2008 [12]	Retrospective study	Dutch	3322	NR	Mixed	Worst PaO_2_	First 24 h	≥120 mmHg (upper quintile)	PaO_2_ between 66 and 80 mmHg	In-hospital mortality
Eastwood 2012 [19]	Retrospective study	Australia	152,680	NR	Mixed	Worst PaO2	First 24 h	≧305 mmHg (upper decile) for adjusted analysis	PaO2 between 75 and 85 mmHg	In-hospital mortality
Elmer 2015 [14]	Reteospective study	USA	184	NR	cardiac arrest	During first 24 h	First 24 h	PaO_2_ > 100 mmHg	PaO_2_ between 60 and 100 mmHg	In-hospital mortality
Fujita 2017 [27]	Reteospective study	Japan		NCT00134472	traumatic brain injury	First PaO_2_	First 24 h	NR	NR	In-hospital mortality
Helmerhors 2015 [21]	Cohort study	Dutch	5258	NR	cardiac arrest	Worst PaO_2_	First 24 h	PaO_2_ > 300 mmHg	PaO_2_ between 60 and 300 mmHg	In-hospital mortality
Helmerhors 2017 [33]	Observational cohort study	Netherland	14,441	NR	post cardiac surgery	Worst PaO_2_	First 24 h	≧200 mmHg	PaO_2_ between 60 and 120 mmHg	In-hospital mortality
Ihle 2013 [15]	Retrospective study	Australia and New Zealand	584	NR	cardiac arrest	Worst PaO_2_	First 24 h	≧300 mmHg	Normoxia	In-hospital mortality
Janz 2012 [22]	Retrospective study	USA	170	NR	cardiac arrest	Highest PaO_2_	First 24 h	≧300 mmHg	Not exposed to hyperoxia	In-hospital mortality
Kilgannon 2011 [23]	Retrospective study	USA	4459		cardiac arrest	First PaO_2_	First 24 h	≧300 mmHg	Not exposed to hyperoxia	In-hospital mortality
Lång 2016 [31]	Retrospective study	Finland	432	NR	hemorrhage	Mean PaO_2_	First 24 h	≧150 mmHg	PaO_2_ between 97.5 and 150 mmHg	3 months mortality
Lee 2014 [24]	Retrospective study	Korea	213	NR	cardiac arrest	Mean PaO_2_	From return of spontaneous circulation to the end of therapeutic hypothermia	≧156.7 mmHg (upper quartile)	PaO_2_ between 116 and 134.9 mmHg (second quartile)	In-hospital mortality
Munshi 2017 [34]	Retrospetive study	Canada	1952	NR	ECMO	First PaO_2_after 24 h	First 24 h	> 100 mmHg	PaO_2_ between 60 and 100 mmHg	In-hospital mortality
Page 2018 [13]	Observational cohort study	USA	668	NR	Mixed	Highest PaO_2_	After intubation	PaO_2_ > 120 mmHg	PaO_2_ between 60 and 120 mmHg	In-hospital mortality
Raj 2013 [26]	Retrospective study	Finland	1116	NR	traumatic brain injury	Worst PaO_2_	First 24 h	> 100 mmHg	Normoxia	In-hospital mortality
Rincon 2014 [28]	Multicenter cohort study	USA	1212	NR	traumatic brain injury	First PaO_2_	First 24 h	≧300 mmHg	Normoxia	In-hospital mortality
Rincon 2014 [29] CCM	Multicenter cohort study	USA	2894	NR	Stroke	First PaO_2_	First 24 h	≧300 mmHg	Normoxia	In-hospital mortality
Russel 2017 [35]	Prospectively study	USA	653	NR	traumatic injuries	Highest PaO_2_	First 24 h	NR	NR	In-hospital mortality
Sutton 2014 [32]	Retrospective study	Australia and New Zealand	83,060	NR	post cardiac surgery	Worst PaO_2_	First 24 h	≧300 mmHg	PaO_2_ between 60 and 300 mmHg	In-hospital mortality
Young 2012 [30]	Retrospective cohort stucy	Australia and New Zealand	2643	NR	stroke	Worst PaO_2_	First 24 h	≧341 mmHg (upper quartile)	Normoxia(PaO_2_ > 69 and < 341 mmHg, 2nd to 9th deciles)	In-hospital mortality

*FiO2* inspired oxygen fraction, *PaO2* arterial partial pressure of oxygen, *SaO2* saturation of oxygen

### Study description

All 24 studies compared the outcomes of hyperoxia with those of normoxia [12–17, 19–36]. The analysis of mortality included 22 studies [12–17, 19–34]. The other two studies were not included in the analysis because of limited number of studies in single primary disease. Details of each study were summarized in Table 1.

### Quality assessment

**Fig. 2:**
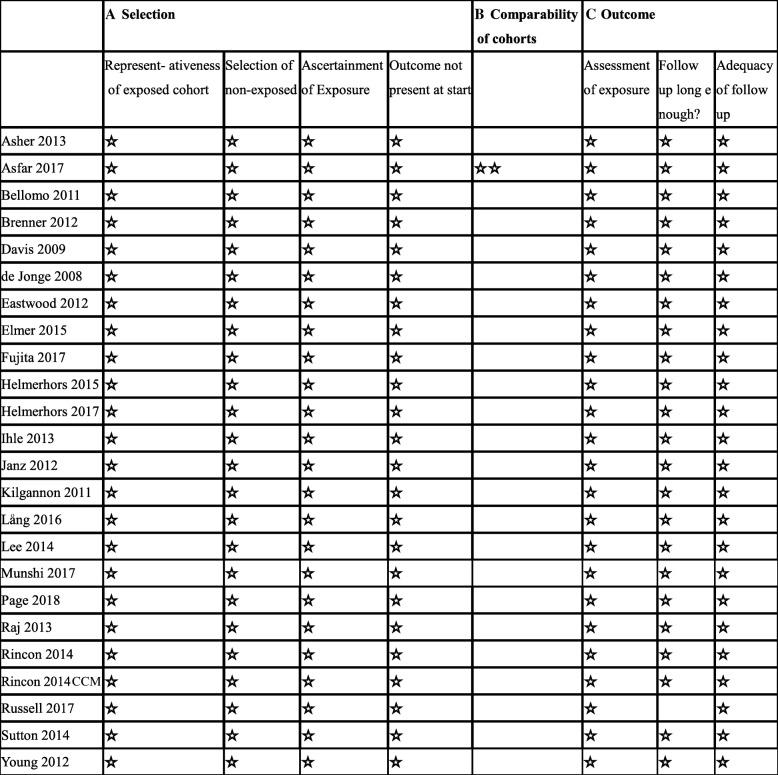
Risk of bias

**Fig. 3:**
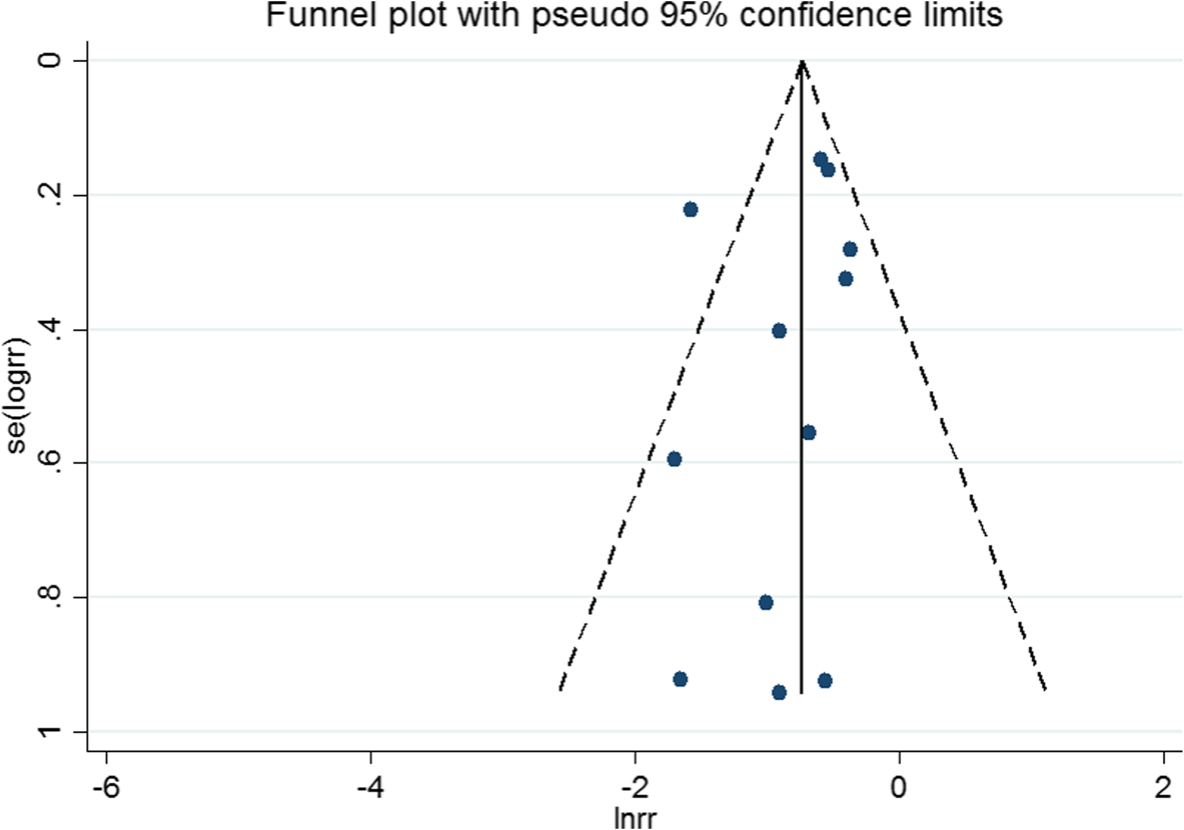
Publication bias

For assessing the risk of bias in the enrolled studies, we used the Newcastle-Ottawa scale. A maximum of 9 points was assigned to each study: 4 for selection, 2 for comparability, and 3 for outcomes. A study with a final score > 6 was regarded as high quality (Additional file 1). Among the 24 studies, one study [36] scored 8 points, 22 studies [12–17, 19–34] scored 7 points, and 1 study scored 6 points [35], which indicated a high risk of bias in the last study (Fig. 2). No studies were excluded for low quality or dubious decisions in the sensitivity analysis. The publication bias was not found (Fig. 3).

### Heterogeneity

No significant statistical heterogeneity was found in mortality between hyperoxia and normoxia groups in patients with stroke and hemorrhage (I^2^ = 48%, *P* = 0.15) or extracorporeal life support (ELS)(I^2^ = 35%, *P* = 0.22). However, significant statistical heterogeneity existed in the comparison in patients with mechanical ventilation (I^2^ = 92%, *P* < 0.01), cardiac arrest (I^2^ = 63%, *P* = 0.01), TBI (I^2^ = 85%, *P* < 0.01) and post cardiac surgery (I^2^ = 80%, *P* = 0.03).

### Mortality

**Fig. 4:**
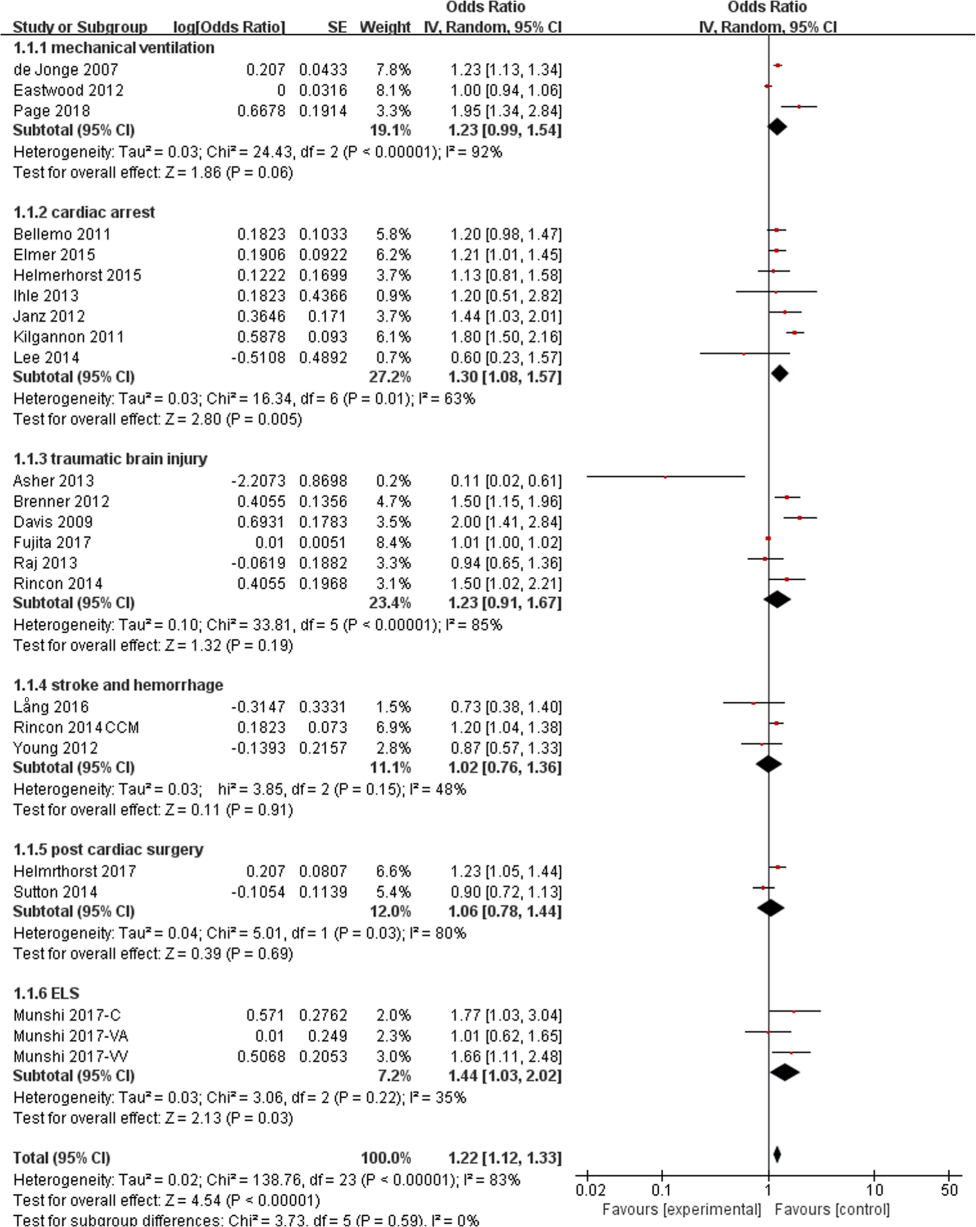
Mortality. OR, odds ratio; CI, confidence interval

Significant difference in the mortality was found between hyperoxia and normoxia groups in patients with cardiac arrest (OR 1.30, 95% CI 1.08~1.57), ELS (OR 1.44, 95% CI 1.03~2.02). However, hyperoxia did not contribute to higher mortality in patients with TBI (OR 1.23, 95% CI 0.91~1.67), stroke(OR, 95% CI), hemorrhage(OR 1.02, 95% CI 0.76~1.36), post cardiac surgey(OR 1.06, 95% CI 0.78~1.44) and mechanical ventilation(OR 1.23, 95% CI 0.99~1.54). Over all, hyperoxia would increase the mortality of patients admitted to ICU(OR 1.22, 95% CI 1.12~1.33). (Figure 4 and Additional files 2, 3, 4, 5, 6 and 7). In addition, 28-day mortality was similar in patients with septic shock between hyperoxia group and normoxia group (hazard ratio 1.27, 95% CI 0.94–1.72; *P* = 0.12) [36]. The same result was also found in the patients with severe traumatic injuries (OR 1.27, 95% CI 0.72–2.25) [35].

## Discussion

In our meta-analysis, we found that hyperoxia would increase the mortality of critically ill patients especially in the ones with cardiac arrest and ELS. In the patients with TBI, stroke and hemorrhage, post cardiac surgery and receiving mechanical ventilation due to mixed primary disease, hyperoxia did not increase mortality.

We found that hyperoxia would lead to adverse outcome in patients with cardiac arrest and ELS. Both of them included a process of reducing blood flow through the cardiac and unstable hemodynamics. The reasons for increased mortality by hyperoxia in patients with these diseases might be as follows. First of all, the exposing to hyperoxia would lead to the increase of reactive oxygen species, which would inhibit the vasodilators such as nitric oxide [37]. Study showed that vasodilator drug-based therapy is a superior solution to reduce mortality in hemodynamically unstable people such as acute myocardial infarction, heart failure or cardiac surgery [38]. Secondly, hyperoxia might increase vascular resistance and reduce cardiac output, coronary blood flow and myocardial O_2_ consumption [5, 6]. Reduced intravascular volume would result in cardiogenic shock, which is a life-threatening condition of circulatory failure [39]. Thirdly, pulmonary toxicity such as endothelial cell destruction, interstitial edema, and type I cell injury caused by the hyperoxia was noticed in animal experiment, and the resulting physiology responses included the increasing of pulmonary leukocyte and neutrophil accumulation, extravascular lung water and permeability surface-area [40]. As a result, the total lung capacity decreased and ventilator-associated lung injury augmented [3, 40]. Clinical study also showed that hyperoxia was associated with higher rate of ventilator associated pneumonia [41]. In addition, oxidative stress due to increased oxygen radical formation is one possible mechanism of ICU-required weakness [42]. Forthly, animal studies showed that high oxygen contraction would increase the oxidative stress and decrease antioxidant. These oxidants can react and damage the cellular macromolecules by virtue of the reactivity that leads to cell injury and necrosis. Oxidants are also mediators in damaging various organs and the organ failure is related to increased mortality [43]. It is reported that a high arterial oxygenation leads to myocardial reperfusion damage, with similar inflammatory consequences secondary to O_2_ free radical development.

However, in patients with TBI, stroke and hemorrhage, post cardiac surgery and received mechanical ventilation due to mixed primary disease, hyperoxia did not influence the mortality. The most possible explanation for this phenomenon is that the number of patients included in each subgroup was too small to find any significant differences in the mortality between the hyperoxia group and normoxia group. However, there are still some assumptions. Traumatic injury and compartment syndrome may appear to be obvious applications for supplementary oxygen because an increased pressure of oxygen would help overcome the decline in perfusion. Several studies have showed the association between hyperoxia and poor outcome after severe TBI [44]. In TBI patients, oxygen delivery can be affected by a decline in cerebral blood flow. In addition, enhancement of brain edema and endothelial swelling after TBI has been demonstrated to decline the diffusion gradient for oxygen to the mitochondria [45]. There are both experimental and indirect clinical studies suggesting that after TBI aerobic metabolism in the brain goes down, [46] which leads to the mitochondrial dysfunction following TBI. Therefore, it is assumed that hyperoxic therapy could improve the oxygen content and thus raise the partial pressure of oxygen, which is the driving force for oxygen move to the mitochondria. This may promote aerobic metabolism in the brain, and thus improves overall outcomes [47]. The same effect also existed in patients with stroke and hemorrhage. There are increasing evidences illustrating that detrimental ischemia-related processes may be present although cerebral perfusion pressure and intracranial pressure levels are adequate [48]. The most effective way to improve hypoxia in brain tissue is to provide higher fraction of oxygen in inspired gas [49]. Therefore, although hyperoxia plays a role in lung injury and various organ failure, the advantages of hyperoxia might overweight the disadvantages and have no effect on the mortality. As for the patients with post cardiac surgery, although no significant difference was found in the mortality, a slight increase in the ICU and hospital stay may indicate a trend towards harm [32]. In addition, type 2 errors and mixed primary diseases may contribute to the result in patients with mechanical ventilation. In patients with septic shock, hyperoxia could lead to vascular contract and reduce oxygen uptake. Thus, hyperoxia could allow for hemodynamic stabilization during vasodilatory shock and do not increase the mortality [50]. Moreover, hyperoxia contributes to the improvement in tissue bed oxygenation in both peri-contusional and remote neuronal tissue, and more aerobic neural metabolic profiles [51]. This might be the reason that hyperoxia did not increase the mortality in patients with severe traumatic injurie.

We should notice that almost all the included studies in our analysis investigated the oxygenation target in the first 24 h rather than the whole phase in the ICU. According to the previous studies, hyperoxia was most common in the first 24 h in ICU. Meanwhile, the lung would get injuries when exposed to hyperoxia within 24 h [52]. In Kraft’s study [53], they investigated the average oxygenation target during the whole ICU stay, which means the high PaO2 happened in the first 24 h might be offset by the low PaO2 value in the following days.

Moreover, clinical heterogeneity existed in our analysis, which might lead to difference in mortality between included studies. First of all, different definition of hyperoxia and normoxia. For example, in the Helmerhors’ study, the definition of normoxia was 60 < PaO2 < 300 mmHg. However, in Elmer’s study, the definition of hyperoxia was PaO2 > 100 mmHg. Thus, part of patients, who should be included in the hyperoxia group in Elmers’ study, were actually included in the normoxia group in the study of Helmerhors. Thus, the application of our study was limited. Secondly, we should notice that demographic information such as age, gender rate and severity of disease of patients were different. For example, the location of arrest was different in the 7 studies, which only included patients with cardiac arrest. The rate of patients’ cardiac arrest happened outside hospital varied from 57 to 100%, which would significantly influence the mortality of patients after cardiac arrest [54, 55].

There are some limitations of our analysis, which should be demonstrated. First of all, almost all the studies included in our analysis were retrospective studies. In this way, we could not figure out the relationship between the severity of the diseases and the level of arterial oxygen pressure. Second, clinical heterogeneity existed due to the mixed definition of hyperoxia, the oxygenation target in comparison group and the timing for measuring the outcome of patients. Third, in some subgroups, the number of patients was too small. Forth, significant statistical heterogeneity existed both in the overall and subgroup comparisons. Thus, the application of our conclusion is limited.

## Conclusions

In conclusion, hyperoxia in patients admitted to the ICU would lead to higher mortality, which has been further confirmed in the patients with cardiac arrest and ELS.

## Additional files

Additional file 1: Assessment of risk of bias and study quality. (DOC 18 kb)

Additional file 2: **Figure S1.** Mortality of patients with mechanical ventilation. OR, odds ratio; CI, confidence interval. (PNG 8 kb)

Additional file 3: **Figure S2.** Mortality of patients with cardiac arrest. OR, odds ratio; CI, confidence interval. (PNG 9 kb)

Additional file 4: **Figure S3.** Mortality of patients with traumatic brain injury. OR, odds ratio; CI, confidence interval. (PNG 9 kb)

Additional file 5: **Figure S4.** Mortality of patients with stroke hemorrhage. OR, odds ratio; CI, confidence interval. (PNG 8 kb)

Additional file 6: **Figure S5.** Mortality of patients with post cardiac surgery. OR, odds ratio; CI, confidence interval. (PNG 7 kb)

Additional file 7: **Figure S6.** Mortality of patients with ELS. ELS, extracorporeal life support; OR, odds ratio; CI, confidence interval. (PNG 8 kb)

## Abbreviations

CENTRAL: Cochrane Central Register of Controlled Trails
CI: confidence interval
ELS: extracorporeal life support
ICU: intensive care unit
ISI: Information Sciences Institute
OR: odds ratio
SD: standard derivation
TBI: traumatic brain injury
