# Supplementary material for: The effect of hyperoxia on mortality in critically ill patients: a systematic review and meta analysis
Source: BMC Pulm Med. 2019 Feb 26;19:53. doi: 10.1186/s12890-019-0810-1 (PMC6390560; doi:10.1186/s12890-019-0810-1)
Supplement: Supplementary file 1 — Assessment of risk of bias and study quality. (DOC 18 kb) [file 12890_2019_810_MOESM1_ESM.doc]

**Additional file 1 Assessment of risk of bias and study quality**

Note: A study can be awarded a maximum of one star for each numbered item within the Selection and Outcome categories. A maximum of two stars can be given for Comparability

**A. Selection**

**1) Representativeness of the exposed cohort**

a) Truly representative of the average cohort of patients with the risk of mortality *

b) Somewhat representative of the average cohort of patients with the risk of mortality *

c) Selected group of users e.g. nurses, volunteers

d) No description of the derivation of the cohort

**2) Selection of the non-exposed cohort**

a) Drawn from the same community as the exposed cohort *

b) Drawn from a different source

c) No description of the derivation of the non-exposed cohort

**3) Ascertainment of exposure**

a) Secure record (e.g. medical records) *

b) Structured interview *

c) Written self report

d) No description

**4) Demonstration that outcome of interest was not present at start of study**

a) Yes *

b) No

**B. Comparability**

**1) Comparability of cohorts on the basis of the design or analysis**

a) Study controls for severity of illness *

b) Study controls for patients of the following: age and co-morbidity *

**C. Outcome**

**1) Assessment of outcome**

a) Independent blind assessment *

b) Record linkage *

c) Self report

d) No description

**2) Was follow-up long enough for outcomes to occur**

a) Yes (hospital stay considered adequate follow up period for outcome of interest) *

b) No

**3) Adequacy of follow up of cohorts**

a) Complete follow up - all subjects accounted for *

b) Subjects lost to follow up unlikely to introduce bias; small number lost to follow-up (> 90 % follow up), or description provided of those lost) *

c) Follow up rate < 90% and no description of those lost

d) No statement
