# Supplementary figures and images for: The effect of hyperoxia on mortality in critically ill patients: a systematic review and meta analysis
Source: BMC Pulm Med. 2019 Feb 26;19:53. doi: 10.1186/s12890-019-0810-1 (PMC6390560; doi:10.1186/s12890-019-0810-1)

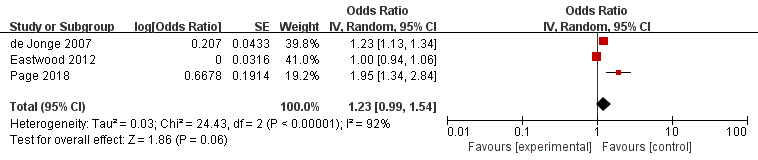

Supplement: Supplementary file 2 — Figure S1. Mortality of patients with mechanical ventilation. OR, odds ratio; CI, confidence interval. (PNG 8 kb) [file 12890_2019_810_MOESM2_ESM.png]

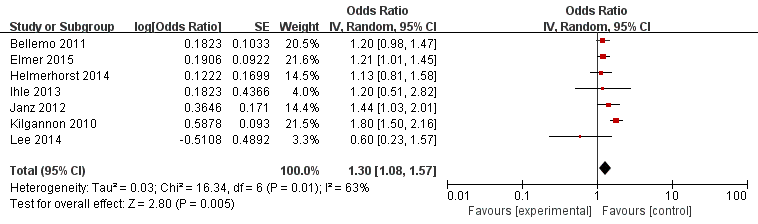

Supplement: Supplementary file 3 — Figure S2. Mortality of patients with cardiac arrest. OR, odds ratio; CI, confidence interval. (PNG 9 kb) [file 12890_2019_810_MOESM3_ESM.png]

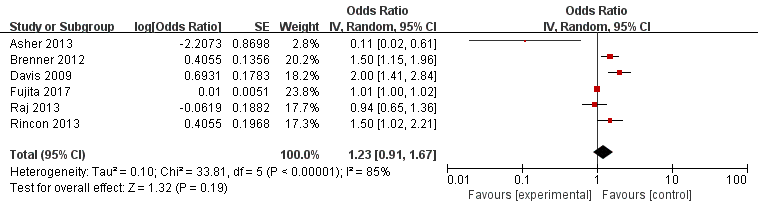

Supplement: Supplementary file 4 — Figure S3. Mortality of patients with traumatic brain injury. OR, odds ratio; CI, confidence interval. (PNG 9 kb) [file 12890_2019_810_MOESM4_ESM.png]

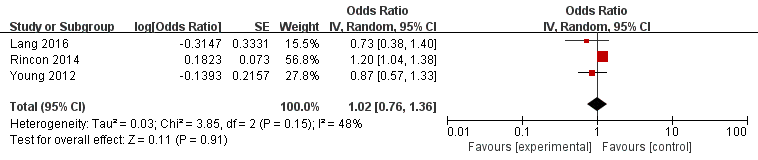

Supplement: Supplementary file 5 — Figure S4. Mortality of patients with stroke hemorrhage. OR, odds ratio; CI, confidence interval. (PNG 8 kb) [file 12890_2019_810_MOESM5_ESM.png]

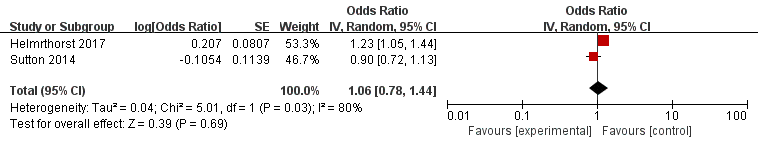

Supplement: Supplementary file 6 — Figure S5. Mortality of patients with post cardiac surgery. OR, odds ratio; CI, confidence interval. (PNG 7 kb) [file 12890_2019_810_MOESM6_ESM.png]

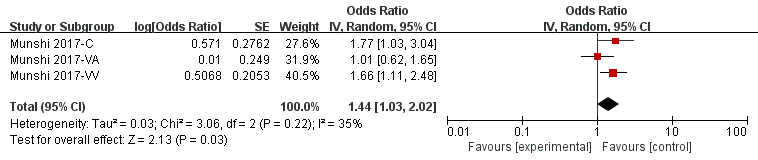

Supplement: Supplementary file 7 — Figure S6. Mortality of patients with ELS. ELS, extracorporeal life support; OR, odds ratio; CI, confidence interval. (PNG 8 kb) [file 12890_2019_810_MOESM7_ESM.png]
